# Multidisciplinary Management of Children with Occult Spinal Dysraphism: A Comprehensive Journey from Birth to Adulthood

**DOI:** 10.3390/children9101546

**Published:** 2022-10-12

**Authors:** Ignazio G. Vetrano, Arianna Barbotti, Alessandra Erbetta, Sabrina Mariani, Stefania M. Bova, Luca Colombo, Valentina Caretti, Federica Marinoni, Elettra Vestri, Giorgio G. O. Selvaggio, Laura G. Valentini

**Affiliations:** 1 Department of Neurosurgery, Fondazione IRCCS Istituto Neurologico Carlo Besta, 20133 Milan, Italy; 2 Dipartimento di Scienze Biomediche per la Salute, Università degli Studi di Milano, 20133 Milan, Italy; 3 Department of Neuroradiology, Fondazione IRCCS Istituto Neurologico Carlo Besta, 20133 Milan, Italy; 4 Pediatric Neurology Unit, Vittore Buzzi Children’s Hospital, 20154 Milan, Italy; 5 Pediatric Orthopedics and Traumatology Unit, Vittore Buzzi Children’s Hospital, 20154 Milan, Italy; 6 Pediatric Surgery Unit, Vittore Buzzi Children’s Hospital, 20154 Milan, Italy

**Keywords:** conus lipoma, multidisciplinary team, occult dysraphism, tethered cord syndrome

## Abstract

Occult spinal dysraphism (OSD) comprises different forms of failure in embryogenic development that can lead to genitourinary, spinal, or lower limb alterations, thus determining progressive neurological deterioration. The correct management of children harboring OSD represents a significant issue during their life up to adulthood. However, patients often have to entertain individual consultations with each specialist. We settled on a multidisciplinary team comprising pediatric neurosurgeons, urologists, neurologists, orthopedists, and other supporting physicians. We present the results of such actions by analyzing a series of 141 children with OSD subjected to neurosurgical procedures, evaluating the impact of multidisciplinary management on outcomes. We also evaluated the specific actions according to the different ages of OSD patients from birth to adulthood to provide a schematic plan that could represent a basis for establishing and disseminating the need for a multidisciplinary approach in OSD management. The multidisciplinary team allows all consultants to see the patient together, covering specific aspects of history and examination pertinent to their management. Offering a one-stop service prevents coordination issues between the different medical teams, avoids delays or cancellations of the various appointments, optimizes cost-effectiveness, and improves efficiency and parents’ satisfaction.

## 1. Introduction

Occult spinal dysraphism (OSD) comprises different forms of failure in embryogenic development that can lead to genitourinary, spinal, or lower limb alterations [1,2]. OSD could determine progressive neurological deterioration secondary to tethered cord syndrome (TCS) [3,4,5]. This complex clinical entity reflects the neuroradiological appearance of the conus, which lies lower than the endplate of L2. The “traction” of the spinal cord can lead to progressive neurophysiological and ischemic alterations occurring with growth and flexion [4]. Nevertheless, acute and sudden neurological deficits are reported, and they can rarely appear as the presenting sign of a previously unknown OSD [6,7]. Finally, this mechanism leads to sensory and motor deficits and urodynamic and anorectal abnormalities; orthopedic deformations may occur too. Moreover, no genetic or metabolic signatures are valid for perinatal screening, not to predict the diseases and outcomes.

Differently from “open” spina bifida [8,9], which shows alterations present at birth and for which there have been significant advances in the genetic and molecular underpinnings of this disease [10], the time and manner in which this spontaneous deterioration manifests in OSD are unknown. There is much controversy regarding whether to perform surgery before or after the onset of such possible deterioration [4,11]. Moreover, tethered cord syndrome and OSD prevalence among children with sacrococcygeal dimples are higher than previously thought [12].

Additionally, the surgical technique is a subject of debate [2], with some groups proposing partial removal [11], recently blamed for carrying a high risk of late deterioration due to re-tethering, with long-term results even worse than natural history [13]. Moreover, the natural history of initially asymptomatic lipomas shows that children with occult dysraphism have a 33% chance of deterioration over nine years [11]. These findings appear similar also in other series, with a deterioration rate up to 40% in ten years [6], with some subgroups as females with transitional lipomas or a terminal syrinx that fared even worse.

Neurological, urological, spinal, and orthopedic deteriorations usually appear as a slow and progressive route, but the onset of such clear and symptomatic manifestations can determine a dramatic worsening only partially reversed by surgical treatment. Therefore, the correct management of children harboring OSD represents a significant issue during their life up to adulthood to prevent and intercept clinical and instrumental signs of such a progressive deterioration. However, in generic hospitals, when the opinion of multiple disciplines is needed to establish a shared plan, patients often have to entertain individual consultations with each specialist, sometimes weeks or even months apart. The multidisciplinary team approach is a well-known issue for open spina bifida cases and is part of specific guidelines [14,15,16]; however, this concept has not yet included OSD due to the more underhand clinical presentation and the debate about the correct management.

We, therefore, set up an organized “one-stop” multidisciplinary clinic for children with OSD scheduled for or subjected to surgery, with all consultations occurring as part of a single appointment. We present the results of such common actions by analyzing an extensive series of children with OSD subjected to neurosurgical procedures, thus evaluating the impact of multidisciplinary management on outcomes. We also evaluated the specific actions of every physician involved in the multidisciplinary team, according to the different ages of OSD patients from birth to adulthood transition, to provide a schematic plan that could represent a base for establishing and disseminating the need for a multidisciplinary approach in OSD management.

## 2. Materials and Methods

We retrospectively reviewed a prospectively collected database dedicated to reporting clinical, surgical, and radiological data on OSD referred to the multidisciplinary outpatients’ team between 2012 and 2022. The team combines neurosurgical expertise from a tertiary, national referral center for elective adult and pediatric neurosurgery (IRCCS Foundation Neurological Institute “Carlo Besta”) with different pediatric specialties from an academic, referral pediatric hospital (Children’s Hospital “Vittore Buzzi”), both in Milan, Italy. All clinical and radiological data from children afferent to the multidisciplinary OSD team during the study timeframe were reviewed and analyzed. We excluded patients with associated anorectal malformations or complex syndromes such as the VACTERL association (vertebral defects, anal atresia, cardiac defects, tracheoesophageal fistula, renal anomalies, and limb abnormalities): the rectal anomalies can negatively impact the final results, via direct damage of sphincters or as a consequence of colorectal surgery. Such a cohort of patients follows a specific integrated pathway, not considered in the present study, with different management [17].

### 2.1. Radiological Assessment

Often, the enrollment is initiated at birth due to the presence in newborns or infants of lumbosacral cutaneous stigmata (including subcutaneous lipomas, naevi, dimples, hair patches, and sinuses), which suggest a possible spinal malformation; ultrasonographic follow-up during pregnancy can also lead to this suspicious. If clinal doubts correctly arise, they usually lead to performing or repeating ultrasonographic examination in the first two months. In cases of documented malformation or uncertain diagnoses, or in older children with cutaneous stigmata or neuro-orthopedic abnormalities that carry a high doubt of OSD, neuroimaging was indicated. MRI is the gold standard for diagnosing OSD, and in our series was performed on all children before surgery, along with spontaneous sleep in newborns up to 3 months. Between this age and five years, the children were sedated, while since the school age, all MRI were executed on awake, collaborative children, often in the prone position to observe the conus movement. The mid-sagittal and axial views on balanced fast field echo (balanced FFE) MRI images or similar high-resolution steady-state gradient-echo sequences were used to determine the level of the conus medullaris and to evaluate the relationship between the lipoma, the conus, and the rootlets, as well as the degree of subcutaneous extension or dermal sinuses.

### 2.2. Neurosurgical Treatment

Our current strategy is to offer prophylactic surgery, ideally at 6–9 months, as it is early enough to avoid neurologic impairment or prevent further deterioration in children with urological impairment. This strategy has the advantage of handling a relatively soft bone and less muscle tissue, facilitating recovery, and allowing a minimally invasive approach with blood loss reduction. In all cases, the neurosurgical approach aims to complete the removal of conus lipomas under intraoperative neurophysiological monitoring (IOM) [18], considering the high recurrence rate after partial removal, reported up to 48% in the first two years [13]. The final goal of surgery is to separate, under microscopic view, the neural placode and all caudal roots (identified through IOM) from the lipomatous tissues to create, through tensionless pia-to-pia neurulation, a neural tube covered by the pial membrane. An expansile duroplasty is performed to prevent re-tethering, creating the so-called “megasac” [19,20]. In the case of fatty filum, surgery is limited to removal of the filum terminalis comprising the localized lipoma, with watertight dural closure but without duraplasty. The spinous process and lamina on both sides, in case of absences of bone cleft, were replaced and secured to the surrounding bones to reduce the risk of spinal alterations during growing.

### 2.3. Urological Assessment

A careful evaluation of voiding habits, physical examination, urinary tract ultrasound, and urodynamic evaluation (UDS) performed following the International Children’s Continence Society recommendation [21] represent the steps of a valuable urological assessment. In pre-toilet-trained patients, UDS included cystometrogram, pelvic floor electromyography using skin electrodes, and abdominal pressure for subtracted detrusor pressure. The UDS was completed with the pressure/flow study in the toilet-trained patients. Video-urodynamic studies require radiological exposure; therefore, they were indicated only in selected cases. After surgery, we repeated urological UDS and urological evaluation at 1, 6, and 12 months and then yearly. Neurogenic detrusor overactivity, detrusor sphincter dyssynergia, reduced or absent bladder sensation, and postvoid residual urine were pathological urodynamic findings.

### 2.4. Neurological Assessment

The neurological examination aims to evaluate muscle tone, strength, and reflexes focusing on the presence of pyramidal signs. Additionally, muscle wasting, foot size disparity, and gait disturbance for older children were taken into high account as possible signs of tethered cord syndrome and neurological deterioration. Moreover, pediatric neurologists and other physicians pay particular attention to the psychological impairment derived from surgery or possible urological and neuro-orthopedic impairment, mainly in adolescent patients. Psychological support is provided to parents (infants and young children) and the children during their growing.

### 2.5. Orthopedic Assessment

Skeletal anomalies represent a relevant marker of symptomatic OSD: particular attention is posed to foot alterations, such as clubfoot, valgus, equinus, calcaneus/calcaneovalgus, talus foot, etc. In selected cases, when necessary, soft-tissue release, different types of osteotomies, or hemiepiphysiodesis for valgus are among the methods used to correct these abnormalities. Finally, severe forms of scoliosis can occur: when indicated, to repair the deformity, reduce morbidity, and prevent its progression in patients with skeletal immaturity, different surgical techniques can be employed according to the degree of scoliosis and the children’s age [22]. Physiotherapy was proposed post-surgically or when indicated during the child’s growth.

## 3. Results

Between 2012 and 2022, 141 children were evaluated by OSD multidisciplinary ODS clinic. Only 110 are still in follow-up because 31 concluded their journey and transitioned to an adult clinic as they turned 18. The age of children at presentation ranged from birth to 18 years, with a mean age of 9.3 years. There were 69 male patients and 72 female patients.

Considering that the short- and long-term results can be secondary to the complexity of the specific dysraphism, we re-analyzed all preoperative MRI, and surgical reports to obtain a uniform classification following Pang’s description [5,19,23,24], which is an overcoming of more dated classification [25], based on the moment of failure during embryonal neural tube development. In our series, the most common diagnosis is filar lipoma FL (n = 56) following by conus lipomas (n = 47), and specifically chaotic (n = 23), transitional (n = 14) and dorsal (n = 8), limited dorsal myeloschisis LDM (n = 22) including two dermoids and two teratomas, retained cord malformation RCM (n = 9), terminal myelocystocele TMC (n = 7), split cord malformation SCM (n = 3). There were also mixed forms: two dorsal lipomas plus RCM, two LDM plus SCM; in eight cases, OSD was associated with Currarino syndrome.

Overall, the majority of patients were subjected to an ultrasound examination at birth due to the presence of cutaneous stigmata. All patients underwent at least a preoperative MRI, usually at 6–12 months; in every case, sedation was necessary to obtain reliable imaging scans. Follow-ups ranged between 6 months and 10 years. However, the mean long-term follow-up (108 months) is long enough to evaluate the long-term effects of the first surgery, whereas in some cases too short to evaluate the risk of re-tethering: in our series, in three cases, re-tethering occurred, 8, 9 and 16 years after the first surgery (1 transitional, 1 chaotic, and 1 LDM, respectively).

Preoperatively, only seven children had lower limb motor deficits; specifically, three FL, two chaotic lipomas, and two transitional lipomas. All of them improved their neurologic function in the immediate postoperative period, except the two children affected by chaotic lipomas. The preoperative UDS showed instead alterations in 63 children. The first clinical urological evaluation was performed in each case except for three (subjected to second surgery for CSF collection), one week after neurosurgical procedures, with clinical and ultrasonographical evaluation. The first postoperative UDS was achieved three months after surgery (early follow-up): considering the 63 patients with preoperative UDS alterations, 18 showed the normalization of UDS parameters, and 45 maintained a stable impairment. Therefore, at late UDS, 11 other children showed the normalization of UDS parameters, while 34 patients confirmed UDS impairment (16 chaotic, 6 LDM, 3 FL, 3 RMC, 3 transitional lipomas, 2 SCM, 1 dorsal lipoma). Unfortunately, 8 out of 78 children with normal preoperative UDS developed postoperative UDS weakening at this early follow-up. For five children, the urological deteriorations were transient, while three children maintained the postoperative UDS impairment, even at the late follow-up. This permanent urodynamic impairment occurred in two LDM and one TMC, which means a surgical deterioration rate of 3.8% (3/78) for the cases at more of a high risk. Three patients started with clean intermittent catheterization (CIC) after surgery, while thirty-six patients continued after surgery with CIC, already commenced before, but progressively reduced the number of such maneuvers per day. Thirty-one patients started anticholinergic medications after surgery once overactivity of the bladder was demonstrated.

In cases of retethering, UDS deteriorated progressively: in one case, a normal sphincter function improved after the second surgery; in another case, the child continued with CIC. The UDS was repeated every one–two years until adulthood, during the growing period, in case of previous and stable impairment for a minority of cases; otherwise, children were followed-up with uroflow and clinical evaluation (see Table 1).

In our series, orthopedic consequences of OSD involved a limited number of patients: specifically, four children went through orthopedic surgery, including two clubfeet and two epiphysiodesis for valgus. Such deformities are related to LDM, chaotic and transitional lipomas. No patients had undergone spine surgery, but 11 children affected by scoliosis were treated with spinal orthoses.

We did not report major surgical complications: cerebrospinal fluid (CSF) collection was noted in nine cases, requiring surgery only in five. In most cases, the cutaneous stigmata short- and long-term cosmetic results were good, whereas in two cases, these results were obtained thanks to the intraoperative assistance of a plastic surgeon. There were no systemic or local infections, but it has to be considered that the healing of cutaneous scar needs much care, with frequent medication to avoid possible contact with urinary and fecal debris. Table 1 summarizes the actions performed by our multidisciplinary team according to the patient’s age.

## 4. Discussion

Establishing a multidisciplinary team to manage OSD allows for a continuous follow-up for children affected from birth (and the diagnosis) to the adult transition. Debates still exist about the diagnosis, surgical indications, treatment, and follow-up. Sometimes the diagnosis is prenatal; however, the presence of cutaneous stigmata at birth represents a warning sign for clinicians that could lead to neuroimaging. The association between complex stigmata or atypical lumbosacral dimples with OSD is well-known, thus suggesting the need for neuroimaging [1].

OSD, mainly spinal lipomas, are progressive diseases. A large French series in 2004 showed that asymptomatic lipomas have a 33% risk of neurological deterioration over 9 years [11]. Similar findings were obtained by an English study [6] in 2012, which reported a 40% deterioration rate in 10 years in unoperated asymptomatic lipomas. Nevertheless, the France group did not favor prophylactic surgery because a partial resection determined a higher probability of late deterioration than lipomas treated conservatively [11]. In contrast, another group showed that gross-total resection, followed by “neurulation” and duraplasty, determines a much better outcome than the natural disease or partial resection [5]: the results seem to confirm that asymptomatic lipomas do indeed need surgery, just not partial resection. This is precisely our experience, considering the rate of persistent impairment at USD in children presenting preoperatively with such alterations. The progression-free probability for total resection is 88.1% at 20 years and 34.6% for partial resection at 10.5 years [24].

However, surgery for OSD, mainly for more complex cases, is not risk-free, including the possible retethering during growth. The benefits of the early radical surgery are obvious in filar, transitional, and dorsal lipomas, or LDM: children within this subgroup showed, after surgery, normalization of pathological preoperative UDS, as also reported by other groups [26]. In contrast, complex forms such as chaotic lipomas presented a high percentage of permanent neuro-urological impairment (80%) without any improvement after surgery. The multidisciplinary approach guarantees, in our opinion, the best strategy to intercept possible early signs of neurological deterioration. Moreover, whereas the role of UDS is undoubtful in managing OSD [25] and suggesting surgery or second operations in late deterioration, urodynamic measurements in children vary widely among pediatric urologists. The interpretation of uroflow curves in children should be cautioned due to possible artifacts secondary to anxiety and the poor cooperation of many of these patients [27]. Analyzing such results in the context of a multidisciplinary team could increase the value of USD results, considering these results as a part of a broader spectrum of diseases that involve the central nervous system and skeletal. Reaching a common decision is sometimes difficult, and this process became easier after a steep learning curve for all involved physicians, who learn from each other to reach a broader, “holistic” vision of the disease course in each child.

Finally, the case manager nurse represents an increased value of a multidisciplinary team, considering the pivotal role in the interaction among parents, physicians, and the children, in sedation for MRI or UDS preparation, coordinating outpatient activities, and suggesting strategies for urinary or bowel management. The case manager has a defined role among individuals with open spina bifida, facilitating access to team-based, patient- and family-centered coordinated care for medical needs [14,28], as also demonstrated in other fields such as oncology [29].

### 4.1. Highlights and Pitfalls during the Children’s Growing

**Figure 1:**
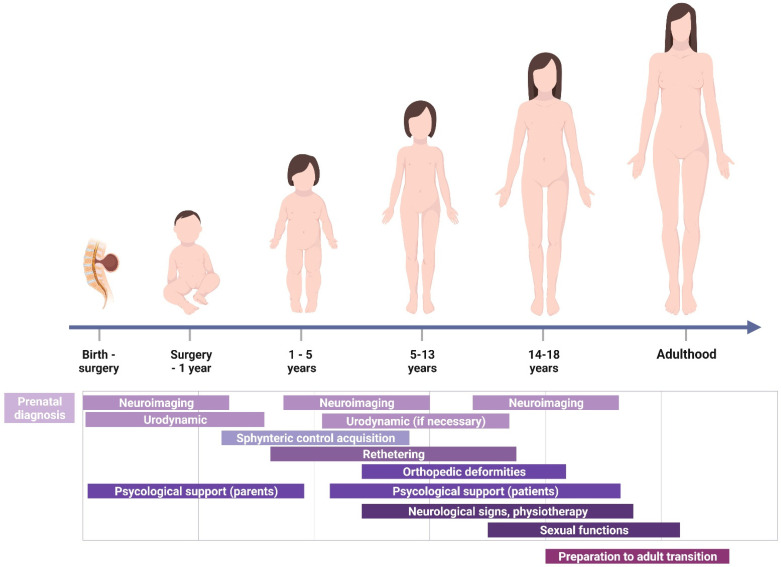
Overview of specific highlights and pitfalls during the children’s growth (created with BioRender.com).

Some indications can be drawn concerning the different clinical points and times of children growing. However, possible acute clinical variation must overcome this more schematic approach, thus anticipating the clinical and instrumental evaluation. Figure 1 shows a graphic render of pitfalls and clinical highlights during growth.

#### 4.1.1. From 0 to 12 Months: Time of the Diagnosis and Surgical Treatment

This time frame is devoted to clinical diagnosis, mainly based on the presence of cutaneous stigmata or after ultrasound evaluation. The MRI confirms or excludes the diagnosis. The urodynamic evaluation is mandatory to assess sphincteric function. The role of neurological evaluation and parent support is limited because it is tough to evaluate the sphincter functions in children younger than three years. Psychological support for parents is also helpful to help in communicating the need for surgical treatment. After surgery, an initial MRI is performed at three months, while UDS at six months. The multidisciplinary team performs a postoperative evaluation just after UDS.

#### 4.1.2. From 1 to 5 Years: Acquisition of Complete Sphincter Control

The attention during this period is related to the evaluation of complete sphincteric control acquisition. Although neurological signs are usually absent, MRI and multidisciplinary clinical evaluation should evaluate possible early retethering. If necessary, physiotherapy is provided, as well as psychological support to parents and, depending on each case, to children. In case of abnormal UDS or children performing CIC, anticholinergic medications or continued antibiotic prophylaxis, if there is evidence of vesicoureteral reflux, are indicated. If necessary, bowel management support is also provided.

#### 4.1.3. From 5 to 13 Years: Orthopedic and Neurological Signs

During this very long period, whereas pyramidal signs are rare, a prone MRI could exclude retethering. Bladder dysfunctions are evaluated, in particular, in children performing CIC. In case of scoliosis or tendon retractions (rare), orthopedic surgeons must decide case by case and consider the advantages and disadvantages of surgical intervention, mainly spinal fusion. Sports activities are recommended. If necessary, psychological support is provided to parents and children.

#### 4.1.4. From 14 to 18 Years: Time of the Diagnosis and Surgical Treatment

Particular attention is necessary at the moment of adult transition. This step still represents a relevant problem not only for children with OSD but also in the case of “open” spinal tube defects [30]. In particular, if a dedicated adult multidisciplinary team is not available, the patients and the parents must be informed that it could be necessary to early organize outpatient appointments with an adult urologist or neurologist with expertise in spinal cord diseases. Sexual dysfunctions must also be considered.

## 5. Conclusions

The multidisciplinary team for OSD allows all consultants to see the patient together, covering specific aspects of history and examination pertinent to their management. Instead of simply coordinating different clinics during the same visit, the combined presence of multiple consultants facilitates communication between specialists and allows all involved specialists to learn from each other. In addition, offering a one-stop service prevent coordination issues between the different medical teams, avoids delays or cancellations of the various appointments, optimizes cost-effectiveness, and improves efficiency and parents’ satisfaction. Considering the specificity of the OSD spectrum and its rarity, high expertise and technical skills are necessary. In our opinion, children with OSD greatly benefit from multidisciplinary management in high-volume referral centers.

## Figures and Tables

**Table 1:** Schematic management of children with occult spinal dysraphism according to the children’s age.

	Urologist/UDS	Pediatric Neurologist	Neurosurgeon	MRI	Orthopedic	Physiotherapy	Psychologist
**Birth–surgery**	6 m	6 m	0–6 m	0–6 m	i.n.	-	parents
**surgery–1 year**	Clinical evaluation after surgery; UDS 3 m, 1 y	3 m, 1 y	3 m, 1 y	1 y	i.n.	i.n.	parents
**1–5 years**	UDS yearly	every 1–2 y	every 1–2 y	i.n.	every 1–2 y	motor scale	parents
**5–13 years**	UroFlowS, UDS if CIC	every 1–2 y	every 1–2 y	Prone, yearly	yearly	i.n.	parents + children
**14–18 years**	UroFlowS, UDS if CIC	every 1–2 y	every 1–2 y	every 1–2 y	yearly	motor scale	patients
**over 18 years**	adult transition	(Adult neurologist i.n.)	if symptoms	i.n.	i.n.	i.n.	i.n.

CIC: clean intermittent catheterization; UDS: urodynamic study; i.n.: if necessary; m: months; y: years.

## Data Availability

The raw data supporting the conclusions of this article will be made available by the authors without undue reservation. Raw data are deposited into an open access dataset (https://zenodo.org).

## Abbreviations

CIC	clean intermittent catheterization
CSF	cerebrospinal fluid
FFE	fast field echo
FL	filar lipoma
IOM	intraoperative neurophysiological monitoring
LDM	limited dorsal myeloschisis
MRI	magnetic resonance imaging
OSD	occult spinal dysraphism
RCM	retained cord malformation
SCM	split cord malformation
TCS	tethered cord syndrome
TMC	terminal myelocystocele
UDS	urodynamic evaluation
VACTERL	vertebral defects, anal atresia, cardiac defects, tracheoesophageal fistula, renal anomalies, and limb abnormalities
